# Emotional School Engagement and Psychiatric Symptoms among 6–9-Year-old Children with an Immigrant Background in the First Years of School in Finland

**DOI:** 10.1007/s10578-020-01086-2

**Published:** 2020-10-26

**Authors:** Heidi Parviainen, Päivi Santalahti, Olli Kiviruusu

**Affiliations:** 1 Department of Public Health, University of Turku, 20014 Turku, Finland; 2 Mental Health Unit, Finnish Institute for Health and Welfare (THL), Helsinki, Finland; 3 Department of Child Psychiatry, University of Turku, Turku, Finland

**Keywords:** Immigrant, Children, School engagement, Mental health

## Abstract

The aim of this study is to examine emotional school engagement and psychiatric symptoms among 6–9-year-old children with an immigrant background (n = 148) in their first years of school compared to children with a Finnish native background (n = 2430). The analyzed data consisted of emotional school engagement measures completed by children and Strengths and Difficulties Questionnaires completed by both parents and teachers. Children with an immigrant background had lower self-reported emotional school engagement than children with a native background with reference to less courage to talk about their thoughts in the class and more often felt loneliness. Further, they reported that they had more often been bullies and seen bullying in the class. Children with an immigrant background had more emotional symptoms and peer problems reported by parents than children with a native background. However, teachers did not report any significant differences.

## Introduction

Recent evidence from the OECD Reviews of Migrant Education shows that immigrant adolescents are at risk of poor school success [1]. School success, in turn, is related to later work success and a lower rate of socioeconomic disadvantages and can thus be described as an indicator of current and future adaptive success of immigrant children [2]. In many cases, problems relating to poorer school success have their origin already in preadolescent years and it has been shown that weak school engagement and poor mental health early in the school career can predict later similar adverse outcomes [3, 4]. Consequently, it is important to improve mental health and strengthen the school engagement of immigrant children as early as possible to avoid possible negative outcomes. The majority of the European studies in the area of school engagement and mental health of immigrant children and adolescents focus on adolescents [5]. However, the first years of school are a crucial developmental period with distinct developmental tasks from those of adolescence [6]. In this article, the term “child” refers to a person under the age of 13 and “adolescent” refers to persons from 13–18 years of age. Despite the aforementioned knowledge, little information is available on school engagement and the mental health of children with an immigrant background in their first years of school in Europe [5].

School engagement is a broad concept that has been analyzed in several ways [7]. One approach is to divide it into emotional, behavioral and cognitive engagement [7, 8]: The emotional engagement describes the extent of children’s positive and negative reactions to school, teacher, and activities. The behavioral engagement encompasses participation in academic activities and conduct. The cognitive engagement generally refers to motivation to master learning tasks. Immigrant adolescents have been shown to have a weaker emotional school engagement compared to natives [9]. For children with an immigrant background, the emotional engagement can be regarded especially crucial considering that children who have positive feelings towards school are likely to succeed better in socio-cultural adaptation and coping with negative emotions [10, 11]. Thus in the present study we focused on the emotional aspect of school engagement.

The emotional school engagement is inversely related to emotional problems [12]. It has been proposed that emotionally engaged adolescents are protected from emotional problems by supportive relationships with teachers and peers [12, 13]. In adolescence, school engagement is generally described to decline during the adolescence years [12, 14], yet the majority of adolescents follow stable trajectories from moderate to very high levels of school engagement [15]. Immigrant adolescents seem to be at particular risk for a declining pattern of school engagement [2, 12]. It has been suggested that immigrant adolescents disengage from school to protect themselves from failures in school success [16].

The previous literature gives mixed results for the mental health status of immigrant children and adolescents. They have been reported to display either more [17–20] or fewer [21–23] psychiatric symptoms than the general population. These two perspectives are called migration morbidity and the immigrant paradox, respectively. From the migration morbidity perspective, immigrants compared to natives display lower mental health and overall adjustment including school success, whereas from the immigrant paradox perspective, immigrants display more positive outcomes than natives [5]. These contradictory results are considered to be associated with differences in migration background, ethnic minority position, cultural background, age, host population, and informants [24, 25]. A recent meta-analysis that combined the results of 51 studies reporting internalizing, externalizing and academic outcomes among immigrant children and adolescents in Europe found that the migration morbidity was better supported than the immigrant paradox [5]. To summarize, immigrant children and adolescents seem to have poorer mental health than natives [17, 18, 26, 27].

Only few European studies have been published on the psychiatric symptoms and the school engagement of children with an immigrant background addressing specifically pre-pubertal school children. In an Italian study, Dimitrova and Chasiotis [28] studied the association of immigrant status with psychosocial adjustment in Albanian and Serbian immigrants compared to Slovene and Italian native children. They found that 7–12-year-old immigrant children in Italy reported lower levels of emotional instability and aggression than native children, but these differences were not reported by teachers. A Swiss study, by von Grünigen et al. [29] showed that 5–6-year-old immigrant children were less accepted by peers and were more often victimized than their Swiss peers. Atzaba-Poria et al. [19] investigated the adjustment of 7–9-year-old Indian children living in Britain. Parents of the Indian children reported more internalizing problems in their children than British parents. No significant differences were found for externalizing or total problem behavior.

Finland has a relatively short history as an immigrant receiving country and the number of immigrants remains small [30]; in 2017, there were 384,000 (7.0%) individuals for whom either both parents or the only known parent had been born abroad [31]. However, Finland is one of the countries where immigrant adolescents are at particular risk of failing to achieve basic academic proficiency: They are more than twice as likely as adolescents without an immigrant background to fail if they have personal migration experience [1]. The adaptation and success of these immigrant generations are important for the development and stability of the country [1, 2]. Considering that adolescents’ academic proficiency is associated with school engagement early on in their school career, it is of utmost importance to study early school engagement in the first years of school [32]. The aim of this study is to examine self-reported emotional school engagement and psychiatric symptoms reported by both parents and teachers among 6–9-year-old children with an immigrant background in the first years of school compared to children with a Finnish native background.

## Methods

### Participants and Procedure

The data used was from a cluster randomized controlled trial of “Together at School” intervention program on children’s socio-emotional skills, carried out by the Finnish Institute for Health and Welfare (THL) during 2013–2014. A detailed description of the trial of “Together at School” intervention program and its data collection methodology has been reported elsewhere [33]. All Finnish primary schools were invited to participate in the study on the condition that the school had a minimum of two teachers who agreed to participate for the whole study period of two school years, and who were teaching the first, second or third grades. The data includes 79 Finnish primary schools with 3704 children [33].

Briefly, the cluster randomized controlled trial of “Together at School” was designed to evaluate children’s socio-emotional skills and mental health as primary outcomes, and related underlying mechanisms along with school and family-related factors as secondary outcomes. The informants consisted of children, parents, teachers and principals. All parents received an information letter regarding the intervention program and the aims of the study. The parents were informed about the voluntary nature of the participation in the data collection and a consent form for data collection was included in the information letter. The teachers and principals consented by agreement. The study protocol of “Together at school” intervention program was approved by the Ethics Committee of the Finnish Institute for Health and Welfare in Helsinki, Finland (27.9.2012) [33].

For the present analysis, the data collected from participants at the baseline in autumn 2013 was analyzed. Parents of altogether 2610 children participated at baseline and of these 2578 provided information on the question of their native language. The analyzed data consisted of the questions of emotional school engagement completed by the children themselves (*n* = 2353) and Strengths and Difficulties Questionnaires (SDQ) completed by both parents (*n* = 2578) and teachers (*n* = 2376). A child with an immigrant background was defined as a child who had at least one parent with a foreign native language. The domestic languages are Finnish and Swedish. Of the 2578 children, 113 (4.4%) had one parent with a foreign native language and 35 (1.4%) had two parents with a foreign native language. A total of 2430 (94.3%) children had both parents with Finnish or Swedish as their native language. There were 974 children in the first grade (37.8%), 999 in the second grade (38.8%) and 605 (23.5%) children in the third grade. In Finland, first to third grade beginners normally cover children from ages 6 to 9 [34].

### Measures

#### Demographic Details

The socio-demographic background information used were gender, school grade, family structure, mother’s basic educational level, parents’ employment status and the family’s self-reported economic situation. Family structure was composed of four categories: nuclear family, single parent family, blended family and other. Family structure was divided into two categories: nuclear family and other. Mother’s basic education was composed of three categories: lower than primary school, primary school and upper secondary school. Mother’s basic education was grouped into two categories: primary school or less and upper secondary school. Parents’ employment status was composed of seven categories: employed, entrepreneur, unemployed, disabled, stay-at-home parent, maternity or nursing leave and student. Parents’ employment status was grouped into two categories: unemployed or disabled and other. Parents reported the family’s economic situation. Parents estimated how easy or difficult it was to cover their living expenses with their income on a six-point scale. The family’s economic situation was grouped into two categories: satisfactory and difficult.

#### Self-reported Emotional School Engagement

Emotional school engagement was measured with a questionnaire specifically developed for the “Together at School” trial to explore the different aspects of emotional school engagement in a way that is comprehensible for small children. The statements of the questionnaire were created based on face validity while including the most widely applied aspects of emotional school engagement [35–37]. The children were asked to answer 14 statements with respect to school on a three-point scale. The statements measured emotional school engagement covering the child’s perceptions of the classroom atmosphere, their relationship with the teacher, their educational achievement, their sense of belonging at school, and bullying (see Table 2).

#### The Prevalence of Psychiatric Symptoms

The SDQ is widely used internationally and consists of a 25-item questionnaire to assess children’s psychiatric symptoms in five sub-scales: hyperactivity, emotional symptoms, conduct problems, problems with peers, and prosocial behavior [38, 39]. Separate questionnaires were completed by both teachers and parents. The SDQ and the background information form were available in Finnish and Swedish, and in the most spoken foreign languages in Finland: Albanian, Arabic, Chinese, English, Estonian, Russian, and Somali [33]. The Finnish version of SDQ has shown adequate psychometric properties [40].

#### Statistical Analysis

Socio-demographic characteristics and emotional school engagement were reported in frequencies separately for the two groups and comparison were tested using Pearson Chi-Square statistic. As the data used was clustered within schools and school class levels, multilevel (mixed) models were used to analyze associations between immigrant background and the outcome variables. The variance component in outcomes due to the school level was shown to be non-significant, and therefore the school level was excluded from the consecutive analysis. Analyses were conducted first with only the immigrant background as the independent variable (univariate) and this was followed by adjusting for socio-demographic background variables (multivariate), including gender, school grade, family structure, mother’s basic education, father’s employment status, mother’s employment status and family’s economic situation. The associations of immigrant background and emotional school engagement have been presented as odds ratios and 95% confidence intervals obtained from generalized linear mixed models. Analyses on parent and teacher-reported SDQ were done using linear mixed models from which the estimated marginal means and their 95% confidence intervals as well as fixed effects estimates for immigrant background have been reported. Regarding the linear mixed models, three (out of 12) analyses had problems in estimation, and these (indicated in Table 4) were analyzed using ANOVA (i.e. not taking into account the clustering of data). The level for statistical significance was set at *p* < 0.05. The statistical analyses were performed with SPSS software version 25.

## Results

### Participants

The background characteristics of the participants are presented in Table 1. There were fewer nuclear families in the immigrant background group (55.4%) compared to natives (76.7%). Parents’ unemployment or inability to work was more common in the immigrant background group among both fathers (11.5%) and mothers (17.6%) compared to natives (5.4% and 6.4%, respectively). Family’s subjective difficulty to cover expenses was greater in the immigrant background group (38.5%) than in the native background group (23.3%).

**Table 1:** Socio-demographic factors in children with an immigrant and a native background

Characteristics	Immigrant background (*n* = 148) %	Native background (*n* = 2430) %	p* (Chi-square)
Gender			0.681
Males	48.0	49.7	
Females	52.0	50.3	
School grade			0.404
1st grade	39.9	37.7	
2nd grade	41.2	38.6	
3rd grade	18.9	23.7	
Family structure			< 0.001
Nuclear family	55.4	76.7	
Other	43.9	23.2	
Missing	0.7	0.1	
Mother’s basic education			0.935
Primary school or less	33.1	33.6	
Upper secondary school	66.2	66.3	
Missing	0.7	0.1	
Parents’ employment status			
Father			0.001
Unemployed or disabled	11.5	5.4	
Other	83.8	93.7	
Missing	4.7	0.9	
Mother			< 0.001
Unemployed or disabled	17.6	6.4	
Other	80.4	93.4	
Missing	2.0	0.2	
Family’s economic situation			< 0.001
Satisfactory	58.1	76.2	
Difficult	38.5	23.3	
Missing	3.4	0.5	

*Between the immigrant background and the native background group

### Emotional School Engagement

The frequencies of responses to the statements of emotional school engagement between the groups are reported in Table 2, and Table 3 shows results from the generalized linear mixed models analyzing these associations. In the unadjusted model, children with an immigrant background had less courage to talk about their thoughts in the class (Disagree *p* = 0.018, Neither agree or disagree *p* = 0.011), felt lonely more often (Disagree *p* = 0.001), had at least one friend in the class less often (Disagree *p* = 0.046) and they had bullied someone in their class more often (Disagree *p* = 0.001, Neither agree or disagree *p* = 0.044). These associations remained significant with the exception of ‘I have at least one friend in the class’ (*p* = 0.065), even when adjusted with socio-demographic factors. Additionally, in the adjusted model children with an immigrant background had more often seen someone in their class being bullied (Neither agree or disagree *p* = 0.049).

**Table 2:** Self-reported emotional school engagement in children with an immigrant and a native background

Statements	Immigrant background (*n* = 133) %	Native background (*n* = 2220)^a^ %	p* (Chi-square)
Classroom atmosphere			
It is peaceful to work in the class			0.651
Agree	36.8	36.6	
Neither agree nor disagree	51.1	48.6	
Disagree	12.0	14.9	
There is good atmosphere in the class			0.850
Agree	62.4	62.7	
Neither agree nor disagree	30.8	31.7	
Disagree	6.8	5.6	
I have the courage to talk about my thoughts in the class			0.019
Agree	32.3	44.8	
Neither agree nor disagree	41.4	34.2	
Disagree	26.3	21.0	
We have fun in the class			0.680
Agree	78.9	78.2	
Neither agree nor disagree	15.8	17.8	
Disagree	5.3	4.0	
Relationship with teacher			
My teacher listens and understands me			0.534
Agree	79.7	83.3	
Neither agree nor disagree	17.3	13.9	
Disagree	3.0	2.8	
I trust my teacher and I can tell him about my things			0.578
Agree	72.9	73.3	
Neither agree nor disagree	24.1	21.9	
Disagree	3.0	4.8	
Sense of belonging at school			
I feel lonely			0.001
Agree	27.8	16.1	
Neither agree nor disagree	18.0	17.2	
Disagree	54.1	66.7	
I have at least one friend in the class			0.074
Agree	68.4	76.3	
Neither agree nor disagree	5.3	5.3	
Disagree	26.3	18.4	
I make friends easily			0.532
Agree	63.9	68.2	
Neither agree nor disagree	30.1	25.7	
Disagree	6.0	6.1	
I get on well at school			0.261
Agree	79.7	75.1	
Neither agree nor disagree	18.0	19.7	
Disagree	2.3	5.2	
Educational achievement			
I do well at school			0.429
Agree	76.7	81.2	
Neither agree nor disagree	21.8	17.4	
Disagree	1.5	1.4	
Bullying			
Someone in my class has bullied me this autumn			0.157
Agree	36.8	30.7	
Neither agree nor disagree	12.0	17.5	
Disagree	51.1	51.8	
I have seen that someone in my class has been bullied this autumn			0.024
Agree	47.4	46.0	
Neither agree nor disagree	25.6	17.6	
Disagree	27.1	36.4	
I have bullied someone in my class this autumn			0.001
Agree	17.3	8.2	
Neither agree nor disagree	9.8	9.7	
Disagree	72.9	82.1	

*Between the immigrant background and the native background group

^a^*n* varies between 2218 and 2220 due to item nonresponse data

**Table 3:** Odds ratios (OR) and confidence intervals (95% CI) of an immigrant background for statements of emotional school engagement from two-level generalized linear mixed models (with child as first level and school class as second level) adjusted with socio-demographic factors

Statements		Unadjusted OR (95% CI)	Adjusted OR^a^ (95% CI)
Classroom atmosphere			
It is peaceful to work in the class	Neither Disagree	1.08 (0.73–1.61) 0.80 (0.44–1.45)	1.10 (0.72–1.66) 0.88 (0.48–1.61)
There is good atmosphere and environment in the class	Neither Disagree	0.99 (0.67–1.48) 1.28 (0.62–2.64)	0.95 (0.62–1.43) 1.17 (0.56–2.45)
I have the courage to talk about my thoughts in the class	Neither Disagree	1.73 (1.13–2.63)* 1.76 (1.10–2.82)*	1.80 (1.17–2.78)** 1.56 (0.96–2.55)
We have fun in the class	Neither Disagree	0.87 (0.53–1.42) 1.35 (0.60–3.01)	0.81 (0.48–1.37) 1.32 (0.58–3.03)
Relationship with teacher			
My teacher listens and understands me	Neither Disagree	1.33 (0.83–2.12) 1.12 (0.40–3.20)	1.33 (0.82–2.17) 0.95 (0.33–2.74)
I trust my teacher and I can tell him about my things	Neither Disagree	1.12 (0.74–1.70) 0.64 (0.23–1.79)	1.13 (0.73–1.76) 0.64 (0.23–1.82)
Sense of belonging at school			
I feel lonely	Neither Disagree	0.63 (0.37–1.07) 0.48 (0.31–0.73)**	0.52 (0.29–0.93)* 0.48 (0.31–0.74)**
I have at least one friend in the class	Neither Disagree	1.14 (0.52–2.52) 1.53 (1.01–2.33)*	1.24 (0.55–2.77) 1.51 (0.97–2.33)
I make friends easily	Neither Disagree	1.24 (0.83–1.84) 1.06 (0.50–2.23)	1.26 (0.83–1.92) 1.07 (0.50–2.29)
I get on well at school	Neither Disagree	0.89 (0.56–1.41) 0.42 (0.13–1.35)	0.85 (0.52–1.39) 0.44 (0.14–1.44)
Educational achievement			
I do well at school	Neither Disagree	1.33 (0.86–2.06) 1.10 (0.26–4.72)	1.36 (0.87–2.13) 0.44 (0.06–3.33)
Bullying			
Someone in my class has bullied me this autumn	Neither Disagree	0.58 (0.33–1.04) 0.83 (0.56–1.23)	0.59 (0.32–1.09) 0.88 (0.58–1.33)
I have seen that someone in my class has been bullied this autum	Neither Disagree	1.41 (0.92–2.18) 0.78 (0.50–1.22)	1.59 (1.00–2.51)* 0.91 (0.58–1.43)
I have bullied someone in my class this autumn	Neither Disagree	0.48 (0.24–0.98)* 0.43 (0.27–0.71)**	0.48 (0.23–1.02) 0.45 (0.26–0.75)**

*p < 0.05, **p < 0.01, ***p < 0.001

The reference category in this analysis is Agree

^a^Adjusting for gender, school grade, family structure, mother’s basic education, father’s employment status, mother’s employment status and family’s economic situation

### Psychiatric Symptoms

Table 4 shows associations between immigrant background and psychiatric symptoms reported by parents and teachers from linear mixed models. Parents in the immigrant background group reported more emotional symptoms (*p* < 0.001) and peer problems (*p* = 0.001) in their children than parents in the native background group. Additionally, parents in the immigrant background group reported higher scores for SDQ total difficulties in their children than the comparison group (*p* = 0.002). In the adjusted models these associations remained significant with the exception of SDQ total difficulties (*p* = 0.088). Teachers did not report any significant differences in psychiatric symptoms between the groups.

**Table 4:** Fixed effect estimates of an immigrant background, and estimated marginal means (M) and confidence intervals (95% CI) for psychiatric symptoms reported by parents and teachers based on Strengths and Difficulties Questionnaire (SDQ) from linear mixed models (with child as first level and school class as second level) adjusted with socio-demographic factors

	Estimated marginal means		Unadjusted		Adjusted	
	Immigrant background M (95% CI)	Native background M (95% CI)	Fixed effect estimate (SE)	*p*	Fixed effect estimate (SE)^a^	*p*
Parents	(*n* = 148)	(*n* = 2430)^b^				
Total difficulties	8.6 (7.8–9.4)	7.2 (7.0–7.4)	1.32 (0.42)	0.002	0.73 (0.43)	0.088
Hyperactivity	3.0 (2.7–3.4)	2.7 (2.6–2.8)	0.29 (0.19)	0.133	0.06 (0.20)	0.766
Emotional symptoms^c^	1.8 (1.5–2.0)	1.2 (1.2–1.3)	0.50 (0.12)	< 0.001	0.35 (0.13)	0.008
Conduct problems^c^	1.8 (1.5–2.0)	1.7 (1.6–1.7)	0.08 (0.13)	0.526	0.02 (0.14)	0.842
Peer problems	2.0 (1.8–2.3)	1.6 (1.5–1.6)	0.45 (0.13)	0.001	0.30 (0.13)	0.026
Prosocial behaviour^c^	7.3 (7.0–7.6)	7.0 (6.9–7.1)	0.30 (0.16)	0.058	0.23 (0.16)	0.152
Teachers	(*n* = 129)^b^	(*n* = 2247)^b^				
Total difficulties	6.3 (5.3–7.2)	6.0 (5.6–6.3)	0.29 (0.50)	0.560	− 0.29 (0.49)	0.558
Hyperactivity	2.6 (2.1–3.1)	2.6 (2.4–2.7)	0.02 (0.25)	0.927	− 0.12 (0.24)	0.620
Emotional symptoms	0.8 (0.6–1.1)	0.9 (0.8–1.0)	− 0.07 (0.13)	0.615	− 0.20 (0.13)	0.139
Conduct problems	1.0 (0.7–1.3)	0.9 (0.8–1.0)	0.06 (0.14)	0.690	− 0.07 (0.14)	0.583
Peer problems	1.8 (1.5–2.1)	1.5 (1.4–1.6)	0.28 (0.15)	0.064	0.11 (0.15)	0.480
Prosocial behavior	6.0 (5.6–6.4)	6.3 (6.1–6.4)	− 0.30 (0.20)	0.146	− 0.28 (0.20)	0.152

^a^Adjusting for gender, school grade, family structure, mother’s basic education, father’s employment status, mother’s employment status and family’s economic situation

^b^*n* varies between 2429–2430, 128–129 and 2246–2247 due to item nonresponse data

^c^Not estimable in the univariate linear mixed model. In multivariate linear mixed model all except emotional symptoms were estimable. The values are from analysis of variance without the clustering effect of school class

## Discussion

This study showed that children with an immigrant background had lower self-reported emotional school engagement than native children with respect to classroom atmosphere and sense of belonging. They had less courage to talk about their thoughts in the class and felt lonely more than the children with a native background. In addition, children with an immigrant background more often reported that they had been bullies and seen bullying in the class. Children with an immigrant background had more emotional symptoms and peer problems reported by parents than children with a native background. Teachers did not report any significant differences in psychiatric symptoms between the two groups. The findings are discussed in detail below.

The results of this study regarding the lower emotional school engagement of children with an immigrant background with respect to classroom atmosphere and sense of belonging are consistent with previous studies on the school engagement of immigrant adolescents. A large review analyzing the emotional and the cognitive school engagement of immigrant adolescents in 41 countries [9], found that immigrant adolescents had weaker emotional school engagement but greater cognitive engagement than native adolescents. Adolescents with better teacher support or classroom climate often had a greater sense of belonging at school and had better attitudes towards school than other adolescents. In a recent OECD review [1], adolescents with immigrant background had a weaker sense of belonging at school. This was influenced by cultural and linguistic differences between the country of origin and the host country. The adolescent’s sense of belonging at school was inversely related to the linguistic distance between the language spoken at home and the language at school [1]. Both peer and teacher support has been shown to have a positive effect on school engagement in younger elementary school children, whereas later in school life, only teacher support is shown to have a positive effect on school engagement [41].

In this study, children with an immigrant background reported that they had more often been bullies and seen bullying in the class than native children. It is important to note that bully and victim roles remain relatively stable from childhood to adolescence [42]. The findings on the association of immigrant or ethnic status and bullying involvement in childhood and adolescence are inconsistent: According to a recent review of Xu et al. [43], some studies have found less bullying perpetration or victimization among immigrants and ethnic minorities, whereas some studies have reported more bullying perpetration or victimization, or stated that being an immigrant does not have an association with bullying involvement. The findings of this study are in line with previous studies showing that immigrant children and adolescents have reported participating in bullying [27, 44, 45]. No significant associations were found between bullying victimization and immigrant background in this study, while previous studies in the early educational [46], and primary school [47] settings in Finland have found more victimization among immigrant children. However, the findings of this study are in accordance with a study among Norwegian youth [44].

With regard to bullying perpetration, it has been proposed that the need for peer acceptance and affiliation is associated with bullying perpetration [44]. Moreover, acculturation stress experienced by immigrant adolescents has been related to physical aggression [48]. Aggression may thus be a response to acculturative stress [49]. However, neither parents nor teachers reported more aggression concerning conduct problems in children with an immigrant background in this study. Understanding the types of bullying perpetration might help to shed light on this finding. The bullying perpetrators can be divided followingly: (1) the aggressive bully is impulsive and aggressive to any person; (2) the anxious bully is insecure and friendless and uses aggression to deal with this stress; (3) the passive bully is aggressive in order to protect himself and to belong to the group [50]. Acculturation stress is suggested to be associated with passive or anxious bullying [51]. The types of bullying perpetration and their associations with the conduct problems may well explain the results showing that children with an immigrant background reported more bullying perpetration than natives, but parents and teachers did not report any differences in conduct disorders between the groups.

This study supports the migration morbidity perspective, with reference to the higher prevalence of emotional symptoms and peer problems reported by parents in children with an immigrant background. In addition, the finding regarding the teacher reports that found no differences in psychiatric symptoms between the groups is in concordance with some previous studies reporting that teachers do not report differences in psychiatric symptoms between native and immigrant children or report less emotional symptoms in immigrant children than parents [23, 52, 53]. The finding of this study correlates favorably with the results of Jäkel et al. [52] who found that when comparing immigrant mothers with German native mothers, Turkish immigrant mothers rated their children’s and adolescents’ total difficulties, emotional symptoms, peer problems and prosocial behavior significantly higher, while there were no differences in the teachers’ ratings between the two groups. In a Dutch study of Vollebergh et al. [23], immigrant parents reported higher internalizing, social and attention problem rates for their daughters than native parents, whereas teachers perceived lower levels of internalizing and social problems especially in immigrant boys, and higher levels of externalizing problems in both immigrant boys and girls. In a Dutch study of Crijnen et al. [53], teachers did not report any differences in mental health between Turkish immigrant and native children and adolescents, but curiously Turkish immigrant teachers reported higher total and internalizing problems for immigrant children and adolescents than the Dutch teachers did. A Finnish study of Säävälä [54] examined school welfare personnel’s and parents’ conceptions of the wellbeing of immigrant children and adolescents and found that they stressed different factors as risks and resources. In views of the school welfare personnel being immigrant in itself does not compose any substantial risk for wellbeing, whereas immigrant parents and native language teachers fear negative attitudes based on ethnic or racial group constitutes a substantial risk for immigrant children’s and adolescents’ wellbeing. Moreover, it has been argued that child health professionals’ identification of psychiatric problems is poorly associated with parent reports regarding economic immigrant children [55]. The findings described here seem to imply that teachers report less psychiatric symptoms than immigrant parents for immigrant children and adolescents, especially in regard of internalizing symptoms. It may be argued that teachers detect immigrant children’s and adolescents’ academic and adaptive problems more easily than their psychiatric problems [56]. Furthermore, one possible explanation for the difference in findings in parent and teacher reports considering emotional symptoms and peer problems could be that children with an immigrant background might actually exhibit problems more differently at home than at school compared to children with a native background.

## Strengths and Limitations

This study adds to the limited body of the European literature of the school engagement and the psychiatric symptoms of children with an immigrant background in the first years of school. The multiple informants (child, parent, teacher) can be regarded as a strength of the study. That the children with an immigrant background formed a heterogeneous group with versatile backgrounds can be seen as a limitation. There are varying unknown reasons behind migration and the type and timing of parental or child’s personal migration and ethnic background all influence the child’s mental health outcome [18, 57]. Also the relatively low number of children with an immigrant background in the sample does not allow analyses in respect of ethnicity. Thus, this study is not comparable to studies focusing on specific immigrant groups. The use of a non-validated questionnaire to assess emotional school engagement can be regarded as a further limitation of the study.

## Summary

The early school engagement and psychiatric symptoms in the first years of school are of great importance given their association to academic proficiency later in school life. This study explored emotional school engagement and psychiatric symptoms among 6–9-year-old children with an immigrant background in their first years of school compared to children with a Finnish native background. The results showed that children with an immigrant background in the first years of school had lower self-reported emotional school engagement and more psychiatric symptoms reported by parents when compared to natives. Teachers did not report any significant differences in psychiatric symptoms between the two groups. Overall, the findings imply that multiple assessment is to be recommended when identifying psychiatric symptoms of young children with an immigrant background. Moreover, these findings seem to highlight the need to establish school-based methods to support the school engagement and the mental health of children with an immigrant background in the first years of school.
